# Impact of Therapeutics on Unified Immunity During Allergic Asthma and Respiratory Infections

**DOI:** 10.3389/falgy.2022.852067

**Published:** 2022-03-25

**Authors:** Armando S. Flores-Torres, Amali E. Samarasinghe

**Affiliations:** ^1^Division of Pulmonology, Allergy-Immunology, and Sleep, Department of Pediatrics, College of Medicine, University of Tennessee Health Science Center, Memphis, TN, United States; ^2^Children's Foundation Research Institute, Le Bonheur Children's Hospital, Memphis, TN, United States

**Keywords:** allergic asthma, respiratory infection, asthma therapy, microbiome, respiratory virus, eosinophils

## Abstract

Asthma is a common chronic respiratory disease that affects millions of people worldwide. Patients with allergic asthma, the most prevalent asthma endotype, are widely considered to possess a defective immune response against some respiratory infectious agents, including viruses, bacteria and fungi. Furthermore, respiratory pathogens are associated with asthma development and exacerbations. However, growing data suggest that the immune milieu in allergic asthma may be beneficial during certain respiratory infections. Immunomodulatory asthma treatments, although beneficial, should then be carefully prescribed to avoid misuse and overuse as they can also alter the host microbiome. In this review, we summarize and discuss recent evidence of the correlations between allergic asthma and the most significant respiratory infectious agents that have a role in asthma pathogenesis. We also discuss the implications of current asthma therapeutics beyond symptom prevention.

## Graphical Abstract

Interplay between infectious agents and allergic milieu. Respiratory infections caused by viruses, bacteria and fungi play an important role in asthma pathogenesis. The immune milieu in allergic asthma may be both defective and protective during respiratory infections. Some bacteria are linked to steroid-resistant neutrophilic asthma and an aberrant immune response. Thermotolerant fungi generally induces a T2 immune response in asthma and are linked to asthma severity and higher corticosteroid requirement. Steroid-resistant neutrophilic asthma is associated with increased airway bacterial burden and reduced bacterial diversity. Corticosteroids and antibiotics induce dysbiosis in asthmatics, which may cause immune system alterations. Biologics and antivirals may be beneficial in some patients. However, the effect of eosinophil depletion on antiviral immunity in asthmatics remains unknown. Influenza and COVID-19 vaccination are recommended in asthmatics, but pneumococcal vaccine benefits are still under debate. 
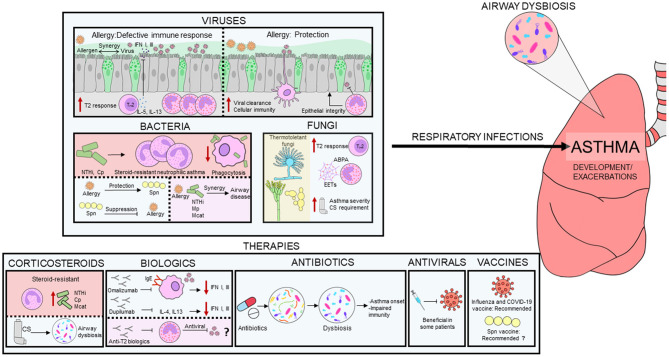

## Introduction

Chronic conditions such as diabetes, obesity, cancer and illnesses affecting the heart, lungs, brain, and kidneys plague modern society. In fact, the Centers for Disease Control and Prevention (CDC) estimate that 60% of adults in the United States have at least one chronic disease while 40% have two or more (1). Afflicting over 300 million people worldwide, asthma is indeed a common chronic condition of the pulmonary system with clear nexus between genetic and environmental factors. The term “asthma” yields >2.5 million hits on Google and over 200,000 articles on PubMed (at writing) indicating that it is a major topic of interest to the lay public and scientists alike. Despite centuries of characterization and garnering knowledge on initiation and pathogenesis of this condition, a cure remains elusive. As a component of the atopic march, asthma develops in early childhood, can affect individuals throughout life (2), and may overlap with chronic obstructive pulmonary disease (COPD) with age (3). Considered a syndrome, asthma is mainly endotyped as type 2 (T2) and non-T2 based on immune bias towards T_H_2-type immune profile. Asthma symptoms allow for further classification based on severity ranging from mild to severe (4). Of the T2 endotype, allergen-induced eosinophilic (allergic) asthma is the most prevalent form resulting from sensitization to airborne environmental allergens, has high incidence, occurs across the ages, and correlates with other chronic conditions like obesity, and therefore will be the focus in this review.

Proposed in 1989, the hygiene hypothesis suggested that early childhood infections are protective against allergic diseases later in life (5). Among the studies of the protective influence of farming exposure in allergy, the Amish and Hutterites studies stand out, as they demonstrate that certain microbial exposures shield against asthma development (6). However, it is evident that not all microbial exposures are protective, as some viruses and bacteria are associated with asthma development instead (7–11).

Indoor and outdoor air are brimming with innocuous and pathogenic infectious agents (12, 13). Despite physical, secreted, and cellular pulmonary defenses in place, some environmental agents infiltrate these safeguards and cause disease (14). This may be particularly problematic in asthma, as the epithelial cell barrier in asthmatics is disrupted, thus facilitating the entrance of allergens and pathogens (15). Moreover, the levels of anti-inflammatory and immunoregulatory factors produced by airway epithelial cells including secretoglobin (SCGB)1A1 are decreased in patients with asthma (16), and airway epithelial cells in asthmatics contain micro-RNA (miRNA) changes implicated in regulation of epithelial cell differentiation (17). The role of the airway epithelium during T2 immune disease has been reviewed extensively (18–20) and therefore will not be the focus of this review.

With anti-inflammatory and pro-reparative functions (21), the T_H_2 immune profile in allergic asthma is commonly considered to be incompetent toward intra- and extra-cellular environmental pathogens like viruses, bacteria, and fungi (22). However, the growing incidence of asthma despite seasonal and pandemic respiratory infections indicate that this immune bias in asthmatics could be either host-protective or pathogen-tolerant. Herein, we explore the literature over the past 15 years focused on the correlations between common respiratory infectious agents and allergic asthma, as hosts with T2 asthma may hold hitherto unidentified anti-pathogen properties that may be of benefit to the modern-day patient.

## Functional Impact of Viral Infections on Asthma Pathogenesis

**Figure 1:**
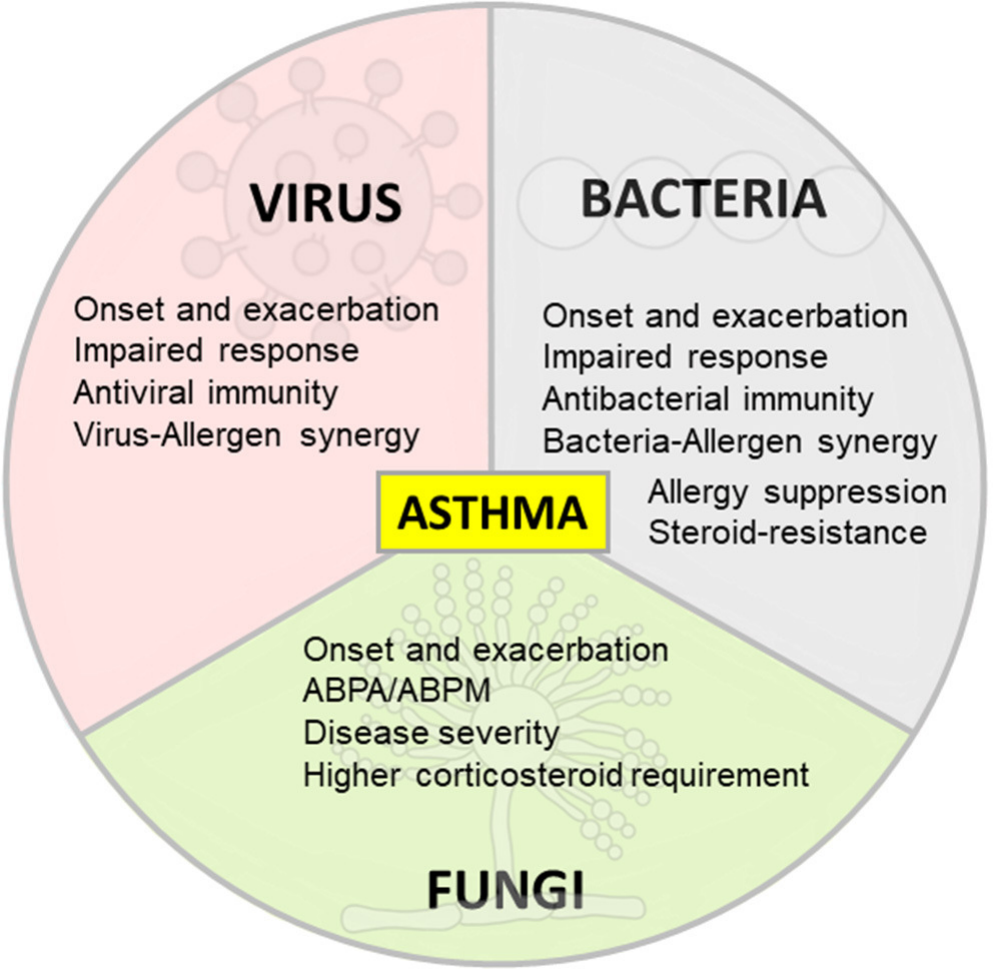
Differing effects of respiratory infections on asthma. Viral, bacterial and fungal respiratory infections are associated with asthma onset and exacerbation. The immune response in allergic asthma is defective against respiratory viruses and bacteria in some scenarios and promote antiviral and antibacterial immunity in other contexts. Allergy and respiratory pathogens may also synergize to increase inflammation and damage in asthma. On the contrary, some bacteria are able to suppress allergic inflammation. Specific bacteria are linked to T2-low asthma, steroid resistance and immune response impairment. Fungal sensitization/infection in allergic asthma is associated with disease severity and higher corticosteroid requirement.

Respiratory viral infections are a leading cause of morbidity and mortality worldwide in pediatric and adult populations with infection severity varying from asymptomatic or mild upper airway infections to bronchiolitis or pneumonia (23). The airway epithelium plays an important role as a first barrier to prevent unrestricted access to environmental pathogens while serving as an initiator of immune responses in the lungs (14, 24). However, allergens and viruses are able to disrupt the epithelial barrier thereby facilitating allergen sensitization and increasing infectious susceptibility (20, 25). In particular, viruses cause junctional protein dysfunction by inducing morphological alterations of epithelial cells such as cytopathic effect or formation of syncytia (18). Respiratory RNA viruses of the families *Paramyxoviridae* (respiratory syncytial virus, parainfluenza virus and metapneumovirus), *Orthomyxoviridae* (influenza virus), *Picornaviridae* (rhinovirus) and *Coronaviridae* (coronavirus) are the most common cause of asthma exacerbations (26), being associated with approximately 80% of exacerbation episodes in both children and adults (27). In the next section we summarize and discuss the current understanding of asthma and its relationship with the most important respiratory viral infections (Figure 1).

### Respiratory Syncytial Virus (RSV)

*Paramyxoviridae* family member RSV, is an enveloped negative-sense single-stranded (ss) RNA virus that invades ciliated bronchial epithelial cells using G and F proteins on its envelope (28). Being a major cause of respiratory tract (RT) infections in pediatric populations, RSV is the most common viral cause of pneumonia with peak incidences during winter (29, 30). Globally, RSV causes 33 million episodes of acute lower respiratory infection, leading to ~3.2 million hospitalizations and up to 200,000 deaths in children <5 years old per year (31). Moreover, many of the admitted children develop acute respiratory distress syndrome (ARDS), an acute life-threatening pulmonary condition (32). Additionally, there is a well-established association between severe RSV bronchiolitis in early life and asthma development in later childhood (7), where both the timing (infection during infancy) and the severity (hospitalization) are important predictors of asthma development (33). This association is more significant in sensitized children, suggesting a synergy between allergy and RSV to promote later asthma (34). However, RSV prevention in healthy preterm infants do not reduce clinically relevant asthma symptoms at 6 years old age (35).

Allergy may induce a defective antiviral immune response in asthmatics as peripheral blood mononuclear cells (PBMC) from asthmatic allergic adults secrete less interferon (IFN)-α compared to healthy controls (36). Furthermore, eosinophils in asthmatics have a reduced ability to bind and possibly inactivate RSV compared to healthy controls (37). In contrast, mouse models have shown a protective role of allergy during RSV infections. *Aspergillus fumigatus*-sensitized and challenged mice infected with pneumonia virus of mice [a virus related to RSV inducing similar pathology as severe RSV infection in children (38)], were protected of lethal infection (39), an effect mediated by recruited eosinophils. Interestingly, OVA-allergic animals were not protected when infected with this virus (39), suggesting that allergen-induced protection from respiratory viral disease may be strongly dependent on immunogenic properties of the allergen.

### Parainfluenza Virus (PIV)

Members of the *Paramyxoviridae* family like PIVs are enveloped, negative sense, ssRNA viruses that utilize hemagglutinin neuraminidase glycoprotein for host cell attachment and F protein for viral fusion (40). PIV has a type-specific pattern of seasonal circulation wherein PIV1 peaks in autumn and PIV3 during the spring-summer (29). PIVs are a major cause of respiratory infections in immunocompromised patients and infants. In fact, PIV is the second most common cause of acute RT infection among children <5 years, just after RSV (41).

Similar to its protective effect during RSV infections, allergy confers host defense against PIV infection. OVA-allergic mice infected with PIV have reduced viral RNA in the lungs compared to non-sensitized mice due to antiviral effects of eosinophilic nitric oxide (42). Nonetheless, as PIVs trigger asthma exacerbations, it is possible that eosinophils, despite their antiviral activity, exaggerate T_H_2 immune responses in asthmatics after PIV infection (42).

### Human Metapneumovirus (hMPV)

Another member of the *Paramyxoviridae* family, hMPV was first isolated in 2001 from children with RT infections (43, 44). The F protein binds to cell surface integrin αvβ mediating membrane fusion (45). With annual rates of hospitalization of 1 per 1000 children <5 years and 3 per 1000 infants <6 months in age, hMPV is a significant health threat to the pediatric population (46). Clinical features of hMPV infection are similar to other respiratory viruses, including upper RT symptoms like rhinorrhea and cough, and lower respiratory illnesses such as pneumonia and bronchiolitis (43).

Similar to RSV, hMPV infections are associated with asthma exacerbations (47) and children hospitalized with hMPV are more likely to have asthma (46). Moreover, hMPV has been linked to asthma development in children (8) and hMPV infection induces longterm pulmonary inflammation and airway hyperresponsiveness (AHR) in experimental models (48). In contrast to reduced IFN production by epithelia from asthmatics in response to human rhinovirus (49), nasal and tracheal epithelial cells from atopic individuals with wheeze or asthma do not display defective type I or III IFN responses after hMPV infection (50, 51). However, nasal epithelial cells from subjects with mild-to-moderate asthma show elevated hMPV replication compared with infection in cells from healthy individuals, a mechanism mediated by apoptosis inhibition via heat shock protein 70 (51).

### Influenza Virus

Influenza viruses are enveloped viruses with a negative sense segmented ssRNA genome, belonging to the *Orthomyxoviridae* family (52). Influenza virus hemagglutinin binds to sialic acid residues on host cells for viral entry, while neuraminidase cleaves sialic acid to release virions (53). Of the four types of influenza virus, A, B, C and D, influenza A virus (IAV) is the most common and pathogenic (54). Annually, IAV causes seasonal epidemics with ~3-5 million cases of severe respiratory illness and around 650,000 deaths worldwide (55), in addition to global pandemics (56).

Allergic inflammation is traditionally associated with influenza immunity. Similar to human rhinovirus (57), plasmacytoid dendritic cells (pDCs) from allergic asthmatics secrete less IFN-α after influenza A or B exposure (58). Furthermore, FcεRI cross-linking on pDCs before virus challenge interferes with IFN-α secretion, TLR7 expression and virus-induced upregulation of pDC co-stimulatory molecules (58). In contrast to the immune protection from seasonal IAV immunized non-allergic mice, immunized allergic mice are susceptible to infection with pandemic IAV (59). However, other experimental and epidemiological studies have shown a different scenario. During the 2009 influenza pandemic, patients with chronic diseases (including asthma) were among the most commonly hospitalized (60) albeit with less severe outcomes related to viral infection compared with non-asthmatics (61). Bronchial epithelia from asthmatics were resistant to IAV-cytopathology and did not have a defective IFN response compared to cells from health donors (62). Experimental models of allergic asthma and influenza have effectively recapitulated that allergic immunity protects the host from severe influenza (62–68). This protective effect has been attributed to enhanced NK cell activation (63), CD8^+^ T cell support provided by eosinophils (62, 65), transforming growth factor (TGF)-β1-induced reduction of inflammation (64), CD11b^+^ DCs (67), and most recently, eosinophil-mediated enhancement of epithelial barrier responses (68). The timing of IAV infection in relation to asthma induction and the state of the allergic airways during infection are crucial for disease outcome. The induction of allergic airway inflammation in formerly IAV-infected mice generated enhanced lung pathology (66) while pre-existing allergic airway inflammation was protective from upcoming IAV infection (66). Infection with IAV during acute allergic inflammation with eosinophilia leads to better outcomes (maintenance of body weight and epithelial barrier and quicker viral clearance) compared to infection during the remodeling phase of allergic asthma (62). These host responses may be site specific as nose-only allergen stimulation and subsequent IAV infection results in increased influenza morbidity and mortality compared to controls (69), a finding that is contrasting to aforementioned studies performed in lower airway inflammation mouse models.

### Human Rhinovirus (hRV)

Genetically classified as types A, B, and C, hRV is a non-enveloped positive sense ssRNA virus belonging to the *Picornaviridae* family (70). hRV-A and hRV-C are particularly relevant as they are more frequent and cause a more severe respiratory illness in infants compared to hRV-B (71). Most hRV-A and -B serotypes bind to intracellular adhesion molecule-1 receptors on host cells for infection; the minor serotypes bind to the low-density lipoprotein receptor (70). On the other hand, hRV-C attaches to cadherin-related family member 3 (72). Clinical symptoms of hRV infection range from asymptomatic and mild self-limiting in immunocompetent hosts, to more severe manifestations such as bronchiolitis and pneumonia in infants and the immunosuppressed (70). While hRVs cause respiratory illness throughout the year they are most frequent during spring and autumn, and disease severity increases in winter (71). Importantly, hRV infection is the most common trigger of viral asthma exacerbations (73), especially hRV-C, which causes the greater number of asthma attacks in children, often with greater severity than hRV-A or hRV-B (74). Furthermore, a clear relationship is established between early life hRV-induced wheezing and asthma development in later childhood (7, 9).

Allergic sensitization may be a risk factor for wheezing during hRV infection (73, 75). Compared to non-atopic asthmatic children, atopic asthmatic children are more likely to present severe viral disease and loss of asthma control after hRV infection (75). Infants and children admitted for wheezing from hRV have higher levels of serum IgE compared to non-wheezing controls and 84% of children with wheezing were sensitized to at least one aeroallergen (73). Furthermore, allergic individuals may have impaired antiviral immunity to hRV. *In vitro* studies have shown that the epithelial inflammatory response to hRV of asthmatics is abnormal and is associated with increased viral replication and virus-induced cytotoxicity (76). Individuals with allergic asthma have baseline differences in gene expression compared to healthy controls including a decreased expression of viral replication inhibitors and gene dysregulation following hRV infection (77). Moreover, patients with atopic asthma have impaired IFN type I and III responses during hRV infection, although not all studies are in agreement (49). Anti-T2 therapies may be beneficial for such patients, to reduce hRV-induced viral exacerbations and restore impaired antiviral responses (49).

### Coronavirus (CoV)

Coronaviruses are enveloped, positive sense ssRNA viruses belonging to the *Coronaviridae* family. The membrane protein (M) and the envelope protein (E) participate in virus assembly, whereas the spike protein (S) mediates viral entry (78). A novel CoV infection outbreak in Wuhan China occurred in 2019 and rapidly spread across the world causing 290 million infections and 5.4 million deaths to date (79), and is now the most severe pandemic of the 21^st^ century. Perhaps because CoVs are able to induce asthma exacerbations (80) and because some asthmatics have deficiencies in antiviral immunity (49), patients with moderate-to-severe asthma were listed at the beginning of the pandemic to be at risk for severe coronavirus disease (COVID) (81). However, the global initiative for asthma (GINA) reported that people with asthma do not seem to have an increased risk of infection from severe acute respiratory syndrome (SARS)-CoV-2, the causative agent of the ongoing COVID-19 pandemic, or present with severe COVID-19 (4). Early findings suggest that like during the 2009 influenza pandemic T2-high asthma endotype may be protective in COVID-19 infection (82). Patients with asthma with absolute eosinophil counts (AEC) ≥150 cells μL are less likely to be admitted (83). Several hypotheses have been presented to date to explain these findings. Patients with allergic asthma have lower expression of angiotensin-converting enzyme (ACE)-2, the primary receptor for SARS-CoV-2 (84), raising the possibility of a reduced risk of SARS-CoV-2 infection. The anti-inflammatory effect of inhaled corticosteroids (ICS) and T2 cytokines have also been proposed as explanations (85). Another possibility is eosinophil antiviral activity (86), which has been reported to other ssRNA respiratory viruses (87, 88). In fact, eosinopenia correlates to poor outcome in patients with COVID-19, and the restoration of eosinophil numbers is linked to disease improvement (89) and T2-low asthma endotype may correlate with severe COVID-19, as IL-17 (a cytokine that participates in T2-low asthma pathogenesis) drives the immunopathogenesis of ARDS in patients with severe COVID-19 (82).

## Impact of Bacterial Infections in Asthma Pathogenesis

Bacterial infections are associated with pathogenesis, exacerbations and chronicity of asthma (90, 91). The knowledge of the relationship between bacteria and asthma continue to grow rapidly, in part due to the utilization of new technologies for bacterial identification, particularly 16S ribosomal RNA gene sequencing (92). Appreciation of the human microbiome, especially the lung microbiome [considered sterile until recently (93)], allowed researchers to recognize the impact of specific bacteria in different pulmonary disorders (94) and their interactions with viral infections in the context of asthma (95). In the following section we discuss the participation of the most significant bacteria linked to asthma (Figure 1).

### *Streptococcus pneumoniae* (Spn)

Pneumococcus is a Gram-positive bacterium with a polysaccharide capsule that plays a critical role in its virulence (96) and is the most common cause of bacterial pneumonia in children (30). Invasive pneumococcal disease [includes bacteremic pneumonia, meningitis, and bacteremia (97)] is a more serious manifestation of Spn infection which usually causes otitis media, sinusitis and bronchitis (98, 99). Antimicrobial resistance in pneumococci is an ongoing problem and about one million children die of pneumococcal disease every year (99).

As a commensal in the upper RT, a large proportion of the population, including asthmatics, carries Spn asymptomatically (98, 100, 101) albeit carriage is more common in asthmatics (101). Infants with higher *Streptococcus* abundance in the nasopharynx are more likely to wheeze at 5 years of age (9, 10). Additionally, Spn infection is linked to asthma exacerbations (102) and its colonization increases in asthmatics that experienced recent exacerbations (103, 104).

Immune responses to Spn in allergic hosts may be age-dependent as neonate Spn-infected mice had elevated airway neutrophils, more severe lung inflammation, enhanced AHR, and increased IL-17A production after OVA sensitization and challenge in adulthood (105). In contrast, Spn infection in adult mice before OVA challenge induces a regulatory T cell (Treg) influx, which correlates with suppression of allergic airways inflammation (105, 106). Infection or treatment with killed Spn or its components reduce OVA-induced eosinophilic inflammation, T2 cytokine release, mucus hypersecretion and AHR (106–110). Furthermore, pneumococcal conjugate vaccine suppressed the critical features of allergic airways inflammation when administered intranasally through induction of Tregs (111) and pharyngeal Spn colonization suppresses the pathophysiology during acute asthma exacerbations in children (112).

Allergic airways inflammation can also play a protective role against Spn lung disease. OVA- or HDM-induced allergic lung inflammation confers protection against an otherwise lethal pulmonary pneumococcal infection with reduced bacterial burden and neutrophils (113). *Aspergillus fumigatus*-induced allergic mice infected with Spn survived the infection as opposed to over 50% mortality in controls potentially through IL-6 regulation of airway barrier integrity (114). In contrast, HDM-induced allergic mice are unable to mount an effective antibacterial response, as allergy impairs neutrophil recruitment resulting in bacterial invasion and dissemination (115). These differences in antibacterial immunity may be due to methodological variations such as the use of different allergen models, different Spn serotypes, and infectious doses and regimens used (116).

### *Haemophilus influenzae* (Hi)

*Haemophilus* species are Gram-negative coccobacilli broadly classified into typeable (encapsulated) and non-typeable (non-encapsulated) strains. Encapsulated bacteria are further subtyped (a through f) by capsule antigenicity (117). While *H. influenzae* type b (Hib) was one of the most frequent causes of lower respiratory infection, its incidence has decreased largely due to vaccination, while that of non-typeable *H. influenzae* (NTHi) has increased (118). A variety of clinical manifestations such as otitis media, sinusitis, conjunctivitis and pneumonia can be caused by NTHi especially in children. Newborns or immunocompromised individuals can also present with invasive infections, including bacteremia and meningitis (119).

As commensals of the lower RT, *Haemophilus* spp. can be found in healthy individuals (120). However, Hi is more frequently associated in patients with asthma (93) and Hi colonization in neonates is a risk factor of asthma development early in life (10). Experimental studies have confirmed this observation wherein NTHi infection in 3-day-old mice increases granulocyte infiltration, elevated mucus production, T2 cytokines and AHR following OVA-challenge (121).

There are multiple associations between NTHi infection and neutrophilic asthma. Infection of mice with NTHi during OVA-induced inflammation suppresses T2-mediated eosinophilic inflammation and while enhancing neutrophilic inflammation through IL-17 (122). Furthermore, the combination of NTHi infection and allergic airways inflammation resulted in a steroid-resistant disease, a feature that resembles neutrophilic asthma in humans (123). In accord, Hi (124, 125) or members of the *Haemophilus* genera (126, 127) have been found in sputum of patients with neutrophilic asthma. Interestingly, the combination of Hi infection and allergic inflammation in mice impairs airway macrophage and neutrophil activation resulting in chronic bacterial infection (123). Similarly, impaired alveolar or monocyte-derived macrophage phagocytosis of Hi has also been reported in patients with severe asthma (128).

### *Moraxella catarrhalis* (Mcat)

As a Gram-negative diplococcus human-restricted commensal of the upper RT, Mcat is pathogenic to both upper and the lower RTs (129, 130). Also functioning as a causative agent of acute otitis media in children, Mcat is a frequent cause of COPD exacerbations in adults (130). Besides its association with asthma development (10), Mcat colonization is linked to loss of asthma control (131) and wheezing episodes (112, 132). Moreover, *Moraxella* is the reported dominant species in nasal passages of children who develop asthma exacerbations, is stably maintained in their airways (133), and found more commonly in acute respiratory infections (9).

Both neutrophilic and eosinophilic inflammation can result after Mcat infection (95). In patients with neutrophilic asthma, high abundance of *Moraxella* and *Haemophilus* taxa have been identified in sputum samples (126, 127). Moreover, sputum neutrophils positively correlate with the relative abundance of *Moraxella* (127), and Mcat colonization have been associated with prolonged and more severe airway obstruction in treatment resistant severe asthma (126). The relative abundance of upper RT *Moraxella* species correlates positively with systemic and airway eosinophilia (134) and elevated levels of eosinophil cationic protein in nasal samples (133). PBMCs from asymptomatic infants that developed asthma by age 7 secrete higher levels of T2 cytokines (IL-5 and IL-13) and IL-17 when exposed to Mcat or NTHi (135), and Mcat promotes IL-8 and IL-33 gene upregulation in A549 human alveolar epithelial cells (133). Infection during HDM sensitization in mice induced airway neutrophilia, eosinophilia, and IFN-γ^+^, IL-17^+^, and IL5^+^/IL13^+^ T CD4^+^ cells, in addition to goblet cell hyperplasia and mucus production compared to allergic mice without Mcat infection (136).

### *Mycoplasma pneumoniae* (Mp)

*Mycoplasma* species possess unique characteristics. They are the smallest prokaryotes, which allow them to pass through cell filters and because they lack cell walls they are insensitive to cellular antimicrobial agents and Gram-staining (137, 138). A member of the *Mycoplasmataceae* family, Mp predominates among disease causing *Mycoplasma* species (138). While Mp infections can occur worldwide at any time of the year, incidence is higher in summer or early autumn (139). Like many other respiratory pathogens, Mp can asymptomatically colonize the RT (140) but have the ability to cause upper and lower respiratory complications including pneumonia (139, 141) and may be responsible for 4–8% of community-acquired bacterial pneumonias, with increases up to 70% during epidemics, affecting groups of all ages, especially children and young adults (141).

The association between Mp and asthma is longstanding where Mp infections have been linked to asthma inception (11) and exacerbations (139). Community-acquired respiratory distress syndrome (CARDS) toxin produced by Mp has been reported to induce allergic airways inflammation in mice, characterized by eosinophilia, mucus production and T2 cytokine secretion (142). Moreover, history of asthma and atopic sensitization are risk factors for refractory Mp pneumonia requiring steroid therapy in children (143) and Mp detection is involved with worsening chronic asthma (140).

Multiple experimental studies have evaluated the role of Mp infection in allergic asthma. Low dose Mp infection enhances IL-4 and eotaxin-2 expression in allergic mice (144) and CARDS toxin exacerbates asthma in OVA-induced allergic mice (145). In contrast, Mp infection before OVA challenge reduces airway mucin secretion through toll-like receptor (TLR)-2/IFN-γ signaling pathway (146). Surfactant protein A (SP-A), the most abundant of the pulmonary surfactant proteins, binds and opsonizes pathogens, including Mp (147). Interestingly, SPA^−/−^ allergic mice infected with Mp have significantly decreased Mp burden compared to controls, a mechanism attributed to eosinophil-mediated killing of Mp, and limited by SPA (148). On the other hand, it has been reported that allergy impairs the immune response against Mp. OVA-allergic mice infected with Mp have higher bacterial burden than non-allergic mice due to inhibition of TLR2 expression and IL-6 production in lung cells (149), and reduced expression of bactericidal/permeability-increasing protein fold-containing family member A1 (150), a protein with antimicrobial properties against bacteria (151) including Mp (152).

### *Chlamydia pneumoniae* (Cp)

The bacterial family *Chlamydiaceae* includes the human pathogen *Chlamydophila pneumoniae*, a Gram-negative obligate intracellular bacteria that is a common cause of acute respiratory infections (153). Pneumonia and bronchitis are the most common clinical manifestations, with approximately 10% of community-acquired pneumonia (CAP) cases and 5% of bronchitis cases (153).

Also associated with asthma exacerbations (154), early-life chlamydial infection enhances allergic characteristics in OVA-sensitized mice (155). Moreover, Cp is found more frequently in asthmatics (156) and the age at which Cp infection occurs seems to be crucial for asthma development as chlamydial infection during early-life (neonatal and infant), but not adult, increases IL-13 expression, mucus-secreting cell numbers and AHR (155). Moreover, infant infection, but not neonatal, increases airway eosinophilia, T2 cytokines and changes in hematopoietic cells, leading to more severe allergic airways disease in later life (155, 157).

Similar to NTHi, Cp-induced airway inflammation is predominantly T2-low. Current chlamydial infection during OVA-induced allergic disease induces neutrophil influx associated with T_H_1/T_H_17 immune responses while attenuating eosinophil recruitment and T2 response (158). Chlamydia-induced severe steroid-insensitive allergic airways disease in mice induces lung mRNA expression of T_H_1 and T_H_17 associated molecules (*Tlr2, Stat1, Ifng, Cxcl9, Cxcl10, Tnf* , *Il17, Il6, Tgfb*, and *Il1b*) and reduction of T_H_2 associated genes (*Il5* and *Il13*) (159). Elevated IL-8 levels and airway lavage fluid neutrophils are reported in Cp positive asthmatic children (160), and Cp infection may drive increased steroid resistance (161).

## Effects of Fungal Sensitization/Infection on Asthma Pathogenesis

As with viruses and bacteria, fungi participate in asthma development and exacerbations (162) despite considerably less information available regarding causation. The fungal microbiome, or mycobiome, has been increasingly appreciated as an important player in health and disease (162, 163), which is evidenced in asthma by the airway fungal alterations in asthmatics compared with healthy subjects (164–166). Fungi that participate in asthma are divided into thermotolerant (allergenic and potentially infectious), and not thermotolerant (mesophilic), which are allergenic but typically not infectious (167). In this segment, we summarize the current understanding of the relationship between the most important thermotolerant fungi and asthma pathology (Figure 1).

### Aspergillus

The genus *Aspergillus* consists of a variety of ubiquitous opportunistic filamentous mold species although only some are human pathogens (168). Inhaled *Aspergillus* conidia are removed by the mucociliary escalator and resident alveolar macrophages in healthy individuals (169). However, *Aspergillus* conidia can germinate and cause invasive infection in immunocompromised individuals (169, 170).

Sensitization to *Aspergillus* species is associated with severe asthma (171, 172), and greater corticosteroid requirement (172). Sensitization to *A. fumigatus* is related to reduced lung function (173), bronchiectasis (174), and asthma exacerbations in children and adults (175). *Aspergillus* is implicated in epithelial barrier impairment as the alkaline protease 1 of *A. fumigatus* causes disruptions between airway smooth muscle cells and extracellular matrix and promotes AHR (176). Host defense against *Aspergillus* in asthmatics is predominantly associated with T2 responses. Mice sensitized and challenged with *A. fumigatus* conidia develop allergic pulmonary inflammation and AHR with elevated serum IgE and pulmonary IL-4 (177), inhalation of *Aspergillus*-associated proteases by naïve mice promotes airway eosinophilia through protease-activated receptor-2 engagement (178), and chitin promotes eosinophil recruitment (179, 180). Consistently, enrichment of *Aspergillus* in the airways is associated with T2-high asthma in humans (166).

Pulmonary aspergillosis can be divided in chronic pulmonary aspergillosis, invasive pulmonary aspergillosis, and allergic bronchopulmonary aspergillosis (ABPA) (181) that principally affects patients with cystic fibrosis and asthma (182). It is estimated that 9% of cystic fibrosis patients (183) and 2.5% of adult asthmatics (184) suffer from ABPA. Furthermore, nearly 35–50% of patients with cystic fibrosis (183) and 24% of patients with severe asthma (185) have sensitization to *A. fumigatus*. Although many fungi are associated with the disease, *A. fumigatus* is by far the most common cause of ABPA (186) due to its marked thermotolerance and small size and surface properties of its conidia that can reach terminal airways (187, 188). The inflammatory response in patients with ABPA is characterized to be T2-biased caused by hypersensitivity to *A. fumigatus*, which includes high levels of total serum IgE and peripheral eosinophilia (189). Eosinophil extracellular traps (EETs) have been identified in bronchial mucus samples of *A. fumigatus* positive asthmatics with ABPA (190, 191). However, since EETs do not affect fungal viability, it is possible that EETs contribute to ABPA pathology by the formation of sticky mucus and granule-mediated epithelial damage (190, 192). In addition, eosinophils contribute to elevated morbidity and decreased *A. fumigatus* clearance to invasive fungal infection in mice (179). In contrast, eosinophil antifungal activity has been demonstrated against *Aspergillus* species in experimental studies such that mouse eosinophils are important contributors of *A. fumigatus* clearance *in vivo* and are able to kill the fungus *in vitro* (193). Similarly, T2 allergic inflammation has a protective role against *A. niger* infection in mice, while *in vitro* experiments showed that eosinophils possess anti-fungal activity (194).

### Penicillium

*Penicillium* species, members of the *Trichocomaceae* family, are common indoor fungi that can be found in a diverse range of habitats (187, 195). Exposure to *Penicillium* has been linked to asthma (187). Increased levels of *Penicillium* is associated with increased exacerbation of current asthma symptoms in children and adults (175). Individuals sensitized to *P. chrysogenum* and *A. fumigatus* have lower lung function compared to those sensitized to *Candida albicans* (a thermotolerant yeast) or non-thermotolerant fungi (196). Additionally, children with asthma sensitized to thermotolerant fungi, including *Penicillium*, have worse lung function, greater systemic corticosteroid requirement, higher total serum IgE and FeNO, and greater sputum eosinophils compared to asthmatic children not sensitized to thermotolerant fungi (197). As such, *Penicillium* species are significantly enriched in patients with asthma, particularly with atopic asthma (166).

### Candida

*Candida* genus is composed of approximately 200 species, only few of them being implicated in human diseases (198, 199). Included in the mycobiome *Candida* is able to colonize the skin, oropharynx, genitourinary and gastrointestinal tracts (187, 198) and when pathogenic (as with *C. albicans*) can quickly progress from superficial mucosal manifestations to life-threatening systemic infections (198, 199). *Candida* species participate in allergic diseases such as atopic dermatitis and asthma (187), are common etiologic agents of allergic bronchopulmonary mycosis (ABPM) (200), and are linked to severe asthma (201).

A high relative abundance of *Candida* in neonatal stool samples is linked to an increased risk of atopy at 2 years and physician-diagnosed asthma at 4 years (202). The use of antibiotics, which has been associated with asthma development (discussed below), triggers bacterial and fungal imbalances resulting in immune dysregulation (162). In an experimental study, antibiotic treatment in allergen-induced airway inflammation resulted in *C. parapsilosis* overgrowth in the gut, which correlated with airway inflammatory cell influx (203). *Candida* overgrowth-induced plasma prostaglandin E2 promotes lung macrophage polarization to M2, resulting in enhanced allergic airway inflammation. Moreover, oral treatment with human-isolated *C. albicans, C. glabrata* or *C. tropicalis* after antibiotic treatment exacerbates airway inflammation (203). In addition to fungal proteases that are known to drive T2 responses and elicit airways disease (204), it was recently demonstrated that *C. albicans* peptide toxin candidalysin is able to induce allergic disease (205). Candidalysin activates platelets stimulating the release of Dickkopf-1 peptide, which in turn coordinates T_H_2 and T_H_17 development during *C. albicans* airway mycosis (205).

### Cryptococcus

*C. neoformans* and *C. gattii* are the etiologic agents of cryptococcosis (206). *Cryptococcus* species possess a polysaccharide capsule that participates in their virulence and differentiate it from other pathogenic yeasts (207, 208). *C. neoformans* has a worldwide distribution and it is disseminated by bird droppings (206, 209). While healthy individuals are able to clear the fungi or establish an asymptomatic infection after inhalation of spores or fungal cells, *Cryptococcus* can either cause pneumonia (210) or disseminate causing conditions such as meningoencephalitis (206) in immunosuppressed patients.

*C. neoformans* can induce a T_H_2 polarization associated with a non-protective antifungal immunity (211, 212). *C. neoformans* infection exacerbates OVA-induced allergic inflammation in rats with increased eosinophils, IgE titers, goblet cells and AHR compared to controls (213). Following infection with *C. neoformans*, IL-13^−/−^ mice show higher survival, lower fungal burden, lower mucus production and reduced AHR compared to IL-13Tg^+^ and wild-type mice (214). On the other hand, IL-4Rα^−/−^ mice are protected to *C. neoformans* infection, depicted by 100% survival compared with 100% mortality of wild-type mice (215). IL-4Rα^−/−^ mice show reduced lung fungal burden compared with controls, in addition to absence dissemination to the brain, and decreased allergic inflammation and AHR (215). Interestingly, it has recently been reported that *C. pseudolongus* is more abundant in patients with asthma than in healthy individuals, and may play a role in asthma pathogenesis, which requires further investigation (216).

## Current Treatment Strategies for Asthma Symptom Prevention and Their Impact on Immunity to Respiratory Infections

**Figure 2:**
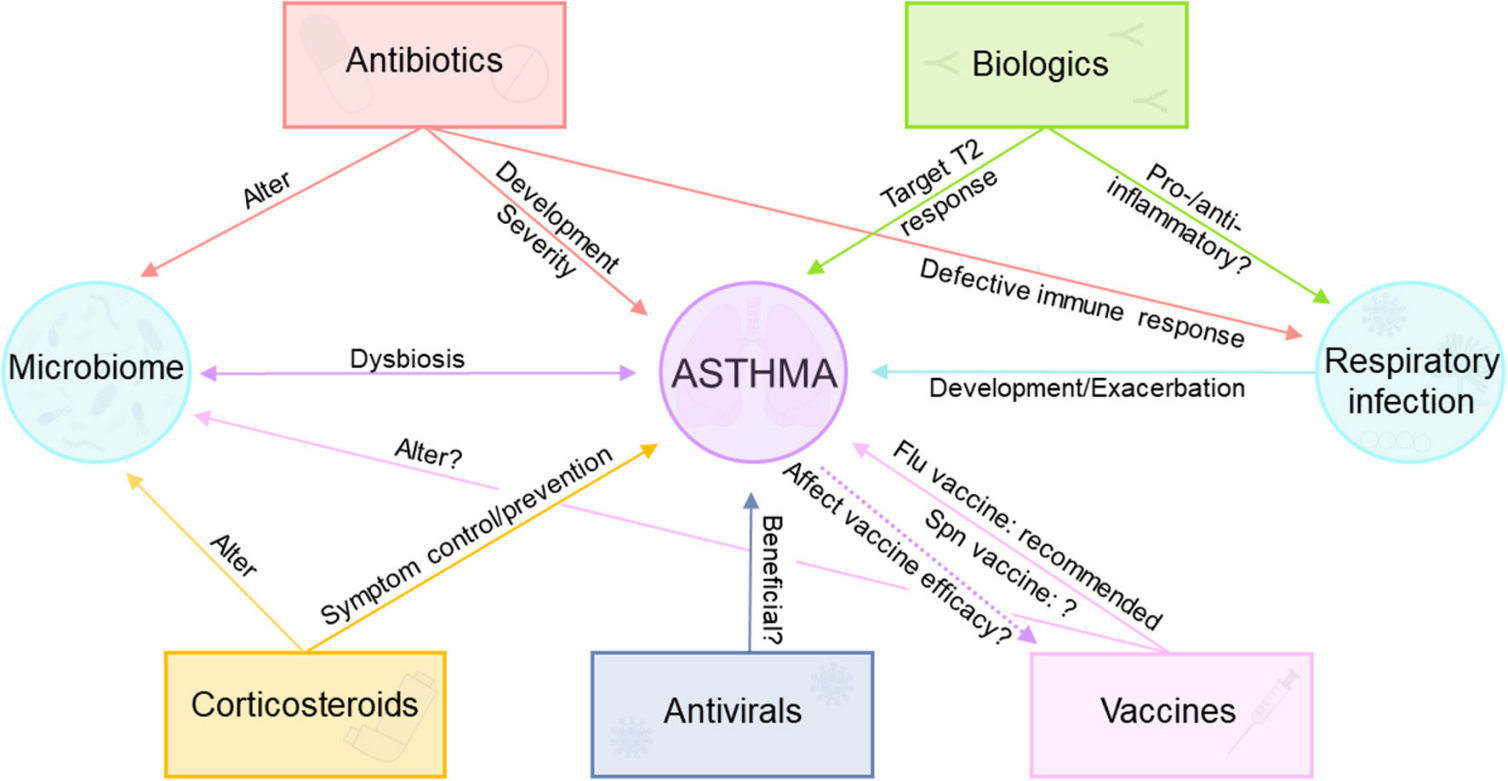
Complex interactions between asthma, asthma therapeutics, and vaccination. Corticosteroids are commonly used for asthma symptom control/prevention, but they alter the airway microbiome. Biologic therapy approved for asthma targets T2 immune response, but their effect during respiratory infections remains unclear. Antivirals may be useful for some asthma patients. Antibiotics cause alterations in the microbiome, are implicated in asthma development and severity, and cause impaired immune responses during viral and bacterial respiratory infections. Influenza vaccine is recommended for patients with asthma, although the usefulness of pneumococcal vaccination is controversial.

Asthma is a multifaceted disease with a varied response to therapy. Although patients with asthma usually respond well to standard therapies, some patients continue to have persistent symptoms (217). Comorbid conditions (e.g. obesity), environmental triggers, and asthma phenotypes are important factors to consider when selecting the optimal therapy for personalized patient care (218, 219). Moreover, increased knowledge about functions of airway and gut microbiota in respiratory diseases (94) obtained in the last decade adds a new level of complexity to effective asthma treatment (Figure 2). In this section we summarize the current known information about asthma therapy and discuss unknowns and areas that could benefit from further research.

### Corticosteroids

The use of inhaled (ICS) or systemic corticosteroids are recommended by the international asthma management guidelines for the control of asthma symptoms and exacerbations (4). In spite of being an effective treatment for most asthmatics, not all patients are responsive. In fact, patients with asthma can be clinically classified depending on the administered CS efficacy into steroid-sensitive and steroid-resistant patients (207). Importantly, CS can cause important adverse effects that can be life-term such as osteoporosis, diabetes, and respiratory infections (220). Furthermore, CS seem to decrease *SCGB1A1* expression (221), and they only modestly correct the alterations of airway epithelial cell miRNA levels found in asthmatics (17). The transglutaminase 2, wingless/integrase 5a and secretory phospholipase A_2_ cascades have been associated with steroid resistance in normal human bronchial epithelial cells and nasal polyp tissues (222), therefore drugs targeting these pathways may be a personalized therapeutic choice for patients with steroid-resistance (222).

Neutrophils, unlike eosinophils, are resistant to CS-induced apoptosis (223), and therefore neutrophilic asthma is steroid-resistant (224). Significant differences in the microbiome of steroid-resistant and -sensitive asthmatics have been reported (225, 226). The baseline composition of bronchial bacterial microbiota from ICS responsive (enriched in *Streptococcaceae, Fusobacteriaceae* and *Sphingomonodaceae*) is different from ICS resistant patients (enriched in *Microbacteriaceae* and *Pasteurellaceae*) and more similar to healthy controls (226). In fact, a member of the Pasteurellaceae family, *Haemophilus*, is linked to steroid-resistant neutrophilic asthma (124, 225). In addition, Hi and Cp, bacteria related to neutrophilic phenotype, drive a steroid-resistant T_H_1/17-associated neutrophilic allergic airways disease in allergen sensitized mice (123, 159, 227). Furthermore, differences in the microbiome between asthma endotypes are reported (226, 228). Patients with neutrophilic asthma have increased airway bacterial burden and reduced bacterial diversity compared to non-neutrophilic asthmatics (228). Similarly, patients with COPD, a chronic disease that in general possess similarities with T2-low asthma phenotype such as airway neutrophilia (229), display reduced bacterial diversity, (230) and an increase in *Haemophilus* and *Moraxella* (231). Conversely, patients with T2-high asthma have lower bronchial bacterial burden than patients with T2-low asthma (226), and lower eosinophil counts are associated with increased airway bacterial load in COPD patients (232).

Augmenting differences in the airway microbiome among asthmatics, CS use is associated with significant changes on the composition (and probably diversity) of the airway microbiome in patients with asthma, COPD and chronic rhinosinusitis (233). Treatment with CS is associated with decreased relative abundance of *Prevotella* species and increased *Pseudomonas* species in asthmatics (234). Children with persistent asthma on regular ICS therapy are nearly four times more likely to have Spn oropharyngeal colonization compared to children not taking ICS (235). Steroid-sensitive asthmatics have an increased relative abundance of *Neisseria* and *Moraxella* species after ICS treatment (226). These studies suggest that CS use can affect the microbiome and neutrophilic inflammation and that the airway dysbiosis and steroid resistance are interrelated. However, as there is considerable heterogeneity between studies with respect to study cohorts, treatment duration, doses and type of evaluated CS (233), further studies are needed to clarify the impact CS have in airway dysbiosis during asthma and other respiratory diseases.

### Biologics

Currently there are five Food and Drug Administration (FDA)-approved drugs – omalizumab (anti-IgE), mepolizumab and reslizumab (anti-IL-5), benralizumab (anti-IL-5Rα), and dupilumab (anti-IL-4Rα) – for moderate to severe asthma that can reduce asthma exacerbations in patients with T2-high asthma (236). These may also be efficacious during viral infections by improving the antiviral response. For example, children with allergic asthma treated with omalizumab have decreased duration and peak level of viral shedding during hRV infections, and ameliorated hRV illness (237). IgE receptor activation increases host susceptibility to viral infection and the use of omalizumab could be beneficial to improve the antiviral response in asthmatics (49). Intriguingly, IgE receptor activation on asthma donor pDCs reduces type I IFN secretion in response to IAV (58), and type I and III IFN release in response to hRV compared to pDCs from non-asthmatics (57). Furthermore, IgE cross-linking on PBMCs exposed to hRV from patients with asthma treated with omalizumab presented an IFN-α increased secretion compared with a placebo group (238). Dupilumab may also improve the antiviral immune response in patients with T2-high asthma, as IL-4 and IL-13 impair the viral-induced interferon production and TLR3 expression (239). In contrast, patients with mild asthma receiving mepolizumab and challenged with hRV have higher viral loads in nasal swabs compared to those that receive placebo, suggesting a protective role of T2 immune response against viral infection (240). This observation, along with the eosinophil antiviral activity against ssRNA viruses demonstrated in experimental studies mentioned above (39, 42, 62, 65, 68), raise the question whether eosinophil-targeted therapies may have a negative impact on viral disease and antiviral host defense. However, currently there are no specific clinical studies that demonstrate that anti-T2 biologics could be detrimental in patients with asthma. Moreover, GINA recommends continuing biologic therapy in patients with severe asthma during the COVID-19 pandemic (4).

### Antivirals

Patients that possess defective IFN responses may benefit from type I and III IFN therapies (241) as these IFNs are able to suppress the T2 responses implicated in asthma (49). In fact, IFN therapies have demonstrated protective roles in experimental models of asthma (241, 242), and a randomized controlled trial suggests that inhaled IFN-β could be beneficial in virus-induced asthma exacerbations in severe asthmatics (243). TLR agonists have also demonstrated positive roles in asthma (244). Resiquimod, a TLR7 agonist, attenuates experimentally-induced allergic inflammation (245) and TLR-9 agonists have demonstrated improvement of asthma symptoms (246). Although palivizumab, a monoclonal antibody against the RSV fusion protein, may reduce subsequent recurrent wheezing in premature infants (247), GINA does not support its use since due to lack of evidence that its effect is sustained (4).

### Antibiotics

Antibiotics are one of the most commonly prescribed medications for children (248), including those with asthma, of which in the United States about 17% of them are prescribed unjustifiably (249). Although antibiotic treatment is crucial against bacterial infections, and may seem attractive for asthma treatment given the implication of bacteria in asthma, they alter the healthy microbiome (250) including the mycobiome (203). This antibiotic-induced dysbiosis, besides the intrinsic gut and lung dysbiosis from asthmatics (94, 251), may cause immune system alterations that are linked to asthma and other pathologies (91, 250). Animal models have supported this concept by showcasing the positive correlation between antibiotic and antifungal treatment and enhanced asthma severity (203, 252–254). Moreover, several studies report an association between prenatal and early life antibiotic exposure in humans and increased risk of asthma development (251, 255–260). Additionally, antibiotic treatment causes longer hospitalization stays and higher costs (261), and have not proven to be beneficial in alleviating asthma exacerbations in adults (262). In fact, the current GINA guidelines do not support the routine prescription of antibiotics to treat asthma exacerbations (unless there is a strong evidence of lung bacterial infection) and recommend avoiding the prescription of broad-spectrum antibiotics during the first years of life (4). Despite this, antibiotics are still commonly used as a general treatment for asthma (263, 264).

Intestinal antibiotic-induced dysbiosis alters immune responses during respiratory bacterial and viral infections. Mice orally treated with broad-spectrum antibiotics and then intranasally infected with Spn have increased lung bacterial burden and accelerated mortality compared with non-treated mice (265). In another study, antibiotic-treated mice displayed impaired innate and adaptive antiviral immunity against IAV infection leading to severe influenza compared to untreated controls (266). Microbe-associated metabolites, like desaminotyrosine and acetate, enhance antiviral responses against IAV and RSV in mice through an upregulation of IFN signaling (267, 268). TLR stimulus provided by bacteria has also been demonstrated to be important to regulate immune responses against respiratory infections like IAV (269). Host protection noted in *A. fumigatus*-sensitized and challenged mice that were co-infected with IAV and Spn is lost after antibiotic-induced airway dysbiosis (270) highlighting the vast impact antibiotics have on pulmonary host defense. Consistent with these studies, children under antibiotic therapy in infancy may have impaired antiviral immunity later in life (271). Furthermore, a reduction of the bacterial genera *Faecalibacterium, Lachonospira, Veillonella*, and *Rothia* has a causal role in asthma development (251). Cumulatively, these studies demonstrate the importance of the gut-lung axis including the microbiome during respiratory infections and highlight the necessity to cease antibiotic misuse and overuse.

Macrolides, one of the most extensively used antibiotics, have been proposed as an attractive therapy for asthma due to its antimicrobial, immunomodulatory and possibly antiviral activities (272). In fact, clinical trials report that azithromycin therapy can reduce asthma exacerbations in adults (273–275), albeit with conflicting data (262) as airway dysbiosis in asthmatics may contribute to asthma pathogenesis (94). Bronchial brushings from asthmatics show a dominance of Proteobacteria, including families of potential pathogens such as *Haemophilus* and *Moraxella* (276), and this phyla was associated with epithelial expression of T_H_17-related genes and worse asthma control (277). A question that still remains is if certain antibiotics are able to equilibrate airway dysbiosis from asthmatics (278), therefore restoring the healthy microbiome. Unfortunately, antibiotics cannot differentiate between commensal and pathogenic bacteria, so pathogen-selective treatments are needed. However, due to antibiotic resistance (279), gut and airway microbiome alterations (280–282), and the aforementioned immunoregulation against respiratory pathogens, antibiotics are not ideal therapies for asthma in the longterm.

### Allergen-Specific Immunotherapy (AIT)

There are a few options for the management of allergic diseases. Excluding allergen avoidance, which is not always practical or possible, conventional pharmacotherapy (e.g. anti-histamines, anti-leukotrienes, CS, etc.) and AIT are the other available options (283). Although pharmacotherapy can control allergic symptoms, they may reappear when medication is interrupted (284, 285). As a treatment based on the administration of increasing doses of clinically relevant allergens over a period of time, AIT represents a promising option through gradual desensitization and/or tolerance (4, 284), therefore providing a longterm solution. The induction of regulatory B cells and Treg and their products (IL-10 and TGF-β) are crucial to obtain tolerance during AIT (284). More recently AIT efficacy was shown to reduce allergic inflammation through *SCGB1A1* induction (286). The first record of AIT is over a century old for the treatment of grass pollen-induced hay fever (287). Since then, the mechanisms behind AIT have been uncovered in some extent, and currently AIT is used for some allergic disorders including rhinitis, venom allergies, and allergic asthma (283). At present, there are two AIT approaches for allergic asthma, which includes subcutaneous immunotherapy (SCIT) and sublingual immunotherapy (SLIT) (4, 288), and SCIT has been shown to successfully alleviate asthma symptoms including bronchial hyperreactivity consequently decreasing the use of asthma medications (288). Although SLIT has demonstrated efficacy as an asthma treatment (289, 290), drawing conclusions is incumbered by the lack of data on outcomes such as exacerbation frequencies, pulmonary functions, quality of life, etc. (291). There are some important disadvantages of AIT such as discomfort from repeated injections, the prolonged time of therapy, lack of commitment in patients, absence of biomarkers that are able to predict the clinical outcome, and importantly, the possibility of life-threatening anaphylactic reactions (283). Additionally, it is important to consider that patients may not feel confident enough to discontinue CS or β-agonist treatments after AIT. Currently, GINA is reviewing evidence regarding AIT as a therapy for asthma, and the next update will cover those findings (4). Therefore, despite its promise as an immunologic solution to asthma, there may be substantial challenges in broad use implementation of AIT as a standard therapy for asthma (283).

## Impact of Vaccinations on Asthma Development/Exacerbation

### Influenza Vaccine

Seasonal influenza vaccines are necessary as circulating influenza strains regularly undergo antigenic drifts. There are two available influenza vaccine formulations in the United States, the inactivated influenza vaccines (IIVs) and live attenuated influenza vaccines (LAIVs) (292). Influenza vaccines are especially relevant during the SARS-CoV-2 pandemic to decrease the burden of respiratory illnesses and avoid hospital saturation (292, 293). Despite this, vaccination coverage in the United States in 2020-21 is nearly 20 percentage points lower than the target of 70% (293). Annual vaccination against influenza virus infection is recommended for all individuals aged ≥6 months who do not have contraindications, especially in populations at higher risk of infection including patients with asthma (4, 294). In fact, asthmatic children are 4-fold more likely to have seasonal influenza-associated hospitalizations than healthy children (295). Influenza vaccination has proven safety and efficacy in asthmatics, as it can reduce the risk of asthma exacerbations, healthcare use, respiratory illnesses, and medications for asthma (296). However, LAIV is contraindicated in wheezing children aged 2–4 years, and precautions should be taken in patients with asthma aged ≥5 years (294) since it was reported to increase the risk of wheezing episodes in infants vaccinated with LAIV compared to IIV (297). However, contraindication of LAIV in asthmatics is not clear since other studies have not reproduced these findings (298–301) and several studies showed that LAIV is safe in children and adults with asthma (301–303).

### Pneumococcal Vaccination

Presently, the 13-valent pneumococcal conjugate vaccine (PCV13) and the 23-valent pneumococcal polysaccharide vaccine (PPSV23) are available in the United States (304). Although the CDC recommend pneumococcal vaccination in asthmatics (305), GINA argues against this due to data insufficiency (4). It has been documented that asthmatics have an increased risk for IPD susceptibility (306) although the influence of the different asthma endotypes is not clear. Interestingly, despite being vaccinated, children with asthma continue to have a higher risk for IPD compared to controls (307). Alternatively, no correlation between IPD-mediated mortality in asthmatics vs. non-asthmatics is reported (308) and asthmatics with CAP have a similar clinical outcome and shorter length of stay compared to the general population (264). Furthermore, asthmatics are not at increased risk of pneumococcal pneumonia hospitalizations compared to COPD patients (309). Recent experimental studies have demonstrated that allergic inflammation confers protection against Spn (113, 114, 270). Notably, the Spn-protected allergic mice displayed reduced levels of pro-inflammatory cytokines and lower levels of airway neutrophils compared to non-protected non-allergic mice (113). Given the heterogeneity of asthma, it is possible that patients with different endotypes show contrasting clinical outcomes to Spn infection. Varying other mechanisms may contribute to these findings. For example, COPD patients [also at high risk for IPD (308)], display reduced bacteria (310) and apoptotic cell (311, 312) phagocytosis. Similarly, macrophages from steroid-resistant severe asthmatics have defects in their phagocytic activity (128), and macrophage efferocytosis is impaired in patients with non-eosinophilic asthma (312). Thus, it is possible that patients with neutrophilic asthma or those on the asthma-COPD spectrum may be at higher risk of bacterial infections.

A subset of asthmatic children with high eosinophil count had poor antibody titers to Spn, even with complete PCV-13 immunization (313). Specific clinical and preclinical studies are necessary to determine the efficacy of pneumococcal vaccines in asthmatics as it is under-investigated, and studies are confounded by differences in age and other susceptibility factors like comorbidity. The need for boosters and their impact on asthma pathogenesis must also be addressed in greater depth. Therefore, the decision to administer pneumococcal vaccines to asthmatics, may need to be done at a more personalized level taking into consideration the type of asthma, other underlying conditions, smoking history, and immunomodulatory therapeutics. It is also important to consider that pneumococcal vaccination has an impact in the airway microbiome as Spn is found as a commensal in healthy subjects, and the eradication of Spn vaccine types may induce airway dysbiosis (314). For example, pneumococcal vaccination increases Hi carriage in healthy children (315, 316), and NTHi-mediated acute otitis media (317). Overall, microbial dysbiosis caused by pneumococcal vaccines is worthy of further investigation.

### COVID-19 Vaccination

At the moment, there are 137 and 194 COVID-19 vaccines in clinical and preclinical development, respectively, and 10 vaccines approved for use by World Health Organization (318) with considerable protection against SARS-CoV-2 infection and disease (319). Currently, COVID-19 vaccination is internationally recommended for asthmatics (4) as vaccination rarely drives allergic reactions (4, 320). However, possible long-term implications of COVID-19 vaccines in asthmatics are currently unknown and require further investigation.

## Conclusion

Immune response “flavor” at steady state is expected to differ between patients living with underlying chronic diseases and healthy hosts. Additional deviations of immune responses are to be expected when considering patients across the age spectrum. Therefore, invariant treatment strategies may not be optimally suited for the modern-day patient. Tests that permit the identification of asthma endotype (blood leukocyte panel, IgE levels, standard cytokine panel to include T_H_1, T_H_2, and T_H_17 cytokines, combined with the allergen identification) should be performed as standard care and results captured in medical records. Considering altered immune responses due to underlying chronic conditions, genetic profiles, and microbial signatures in addition to current parameters of age, sex, and race, hold promise to improve individualized patient care. Incorporating information regarding immune response attributes into standard treatment protocols may help reduce the overuse of immune-altering medications such as antibiotics and corticosteroids. Host-pathogen interactions that occur in patients with underlying allergic asthma when infected with common airborne pathogens are complex, multifaceted, and context dependent. Therefore, targeted studies are necessary to profile these patients for treatment regimens to influence and improve personalized, efficient healthcare with reduced drug burden during respiratory infections.

## Author Contributions

The article was conceived by AES who also wrote the Introduction and conclusion. ASF-T wrote the first draft and drew the figures. Both authors participated in editing the paper and approved the final submission.

## Funding

The Samarasinghe group supported in part by the National Institute of Allergy and Infectious Diseases of the National Institutes of Health under the Award number R01 AI125481 to AES, the Plough Foundation to AES, and the American Lung Association to AES.

## Author Disclaimer

The content is solely the responsibility of the authors and does not necessarily represent the official views of the National Institutes of Health.

## Conflict of Interest

The authors declare that the research was conducted in the absence of any commercial or financial relationships that could be construed as a potential conflict of interest.

## Publisher's Note

All claims expressed in this article are solely those of the authors and do not necessarily represent those of their affiliated organizations, or those of the publisher, the editors and the reviewers. Any product that may be evaluated in this article, or claim that may be made by its manufacturer, is not guaranteed or endorsed by the publisher.
